# Hierarchical combinatorial deep learning architecture for pancreas segmentation of medical computed tomography cancer images

**DOI:** 10.1186/s12918-018-0572-z

**Published:** 2018-04-24

**Authors:** Min Fu, Wenming Wu, Xiafei Hong, Qiuhua Liu, Jialin Jiang, Yaobin Ou, Yupei Zhao, Xinqi Gong

**Affiliations:** 1 Mathematics Department, School of Information, Renmin University of China, Beijing, China; 2 Mathematical Intelligence Application Lab, Institute for Mathematical Sciences, Renmin University of China, Beijing, China; 3 Department of General Surgery, Peking Union Medical College Hospital, Chinese Academy of Medical Sciences and Peking Union Medical College, Beijing, China

**Keywords:** Pancreas segmentation, Single object segmentation, Multi-layer up-sampling structure

## Abstract

**Background:**

Efficient computational recognition and segmentation of target organ from medical images are foundational in diagnosis and treatment, especially about pancreas cancer. In practice, the diversity in appearance of pancreas and organs in abdomen, makes detailed texture information of objects important in segmentation algorithm. According to our observations, however, the structures of previous networks, such as the Richer Feature Convolutional Network (RCF), are too coarse to segment the object (pancreas) accurately, especially the edge.

**Method:**

In this paper, we extend the RCF, proposed to the field of edge detection, for the challenging pancreas segmentation, and put forward a novel pancreas segmentation network. By employing multi-layer up-sampling structure replacing the simple up-sampling operation in all stages, the proposed network fully considers the multi-scale detailed contexture information of object (pancreas) to perform per-pixel segmentation. Additionally, using the CT scans, we supply and train our network, thus get an effective pipeline.

**Result:**

Working with our pipeline with multi-layer up-sampling model, we achieve better performance than RCF in the task of single object (pancreas) segmentation. Besides, combining with multi scale input, we achieve the 76.36% DSC (Dice Similarity Coefficient) value in testing data.

**Conclusion:**

The results of our experiments show that our advanced model works better than previous networks in our dataset. On the other words, it has better ability in catching detailed contexture information. Therefore, our new single object segmentation model has practical meaning in computational automatic diagnosis.

## Background

Recently, due to the great development in deep neural network and increasing medical needs, Computer-Aided Diagnosis (CAD) system has become a new fashion. The high morbidity of pancreas cancers leads to great interest in developing useful CAD methods for diagnosis and treatment, in which accurate pancreas segmentation is fundamentally important. Therefore, developing an advanced pancreas segmentation method is necessary.

Nowadays, pancreas segmentation from Computed Tomography (CT) images is still an open challenge. The accuracy of pancreas segmentation in CT scans is still limit to 73% Dice Similarity Coefficient (DAC) on the patients without pancreatic cancer lesion [1–6], since the pancreas with cancer lesion are more challenging to be segmented. Previous efforts in pancreas segmentation are all referred as MALF (Multi-Atlas Registration & Label Fusion), a top-down model fitting method [1–4]. To optimize the per-pixel organ labeling process, they are all based on applying volumetric multiple atlas registration [7–9] and robust label fusion approach [10–12].

Recently, a new bottom-up pancreas segmentation method [5] has been reported, based on probability maps, which are aggregated to classify image regions, or super-pixels [13–15], into pancreas or non-pancreas label. By leveraging mid-level visual representations of image, this method aims to enhance the segmentation accuracy of highly deformable organs, such as the pancreas segmentation. Furtherly, this work has been improved [6] by using a set of multi-scale and multi-level deep Convolutional Neural Networks (CNN) to confront the high complexity of pancreas appearance in CT images.

In the past few years, deep CNN has become popular in the computer vision community, owing to its ability to accomplish various state-of-the-art tasks, such as image classification [16–18], semantic segmentation [19, 20] and object detection [21–24]. And there is a recent trend of applying it in edge detection, object segmentation and object detection [25] in medical imaging, and a series of deep learning based approaches have been invented. Fully Convolution Network (FCN) [20] adopts a skip architecture combining information from a deep layer and a shallow layer, which could produce accurate and detailed segmentations. Besides, the network could take input in arbitrary size and produce correspondingly-sized output. Holistically-nested edge detection (HED) [26] has been developed to perform image-to-image training and prediction. This deep learning model leverages fully convolutional neural networks and deeply-supervised nets, and accomplishes the task of object boundary detection by automatically learning rich hierarchical representations [17]. In the observation that only adopting the features from the last convolutional stage would cause losing some useful richer hierarchical features when classifying pixels to edge or non-edge class, richer convolutional features network (RCF) has been developed. Combining the multistage outputs, it accomplishes the task of edge detection better.

However, when it comes to the single object segmentation (pancreas segmentation), the RCF does not achieve great performance as in edge detection, because the detailed texture information of the object caught by the network is not accurate enough. To overcome this difficulty, we propose a novel multi-stage up-sampling structure into the network, to accomplish the task of single object segmentation (pancreas segmentation) more perfectly. In the following method section, we will explain our dataset, the detail of the multi-layer up-sampling structure,the loss function we used, the whole workflow, and the evalution criteria.Besides, the experiment result will be shown in the results section.

## Methods

### Dataset

**Fig. 1:**
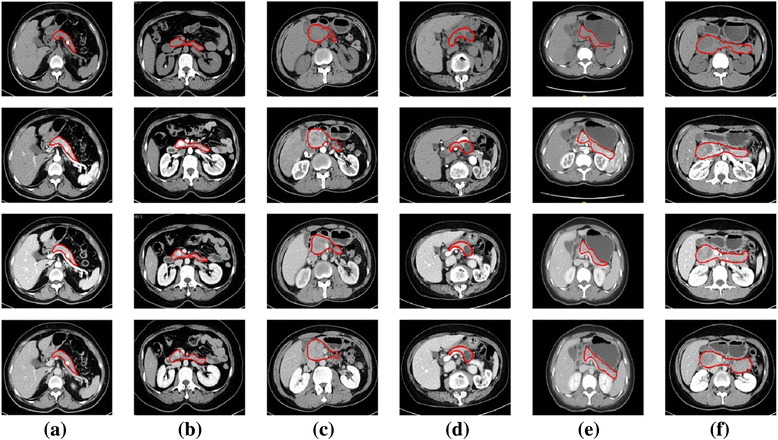
Examples of the six types (including non-disease) of abdomen CT image for (**a**) Healthy, (**b**) PNET, (**c**) PDAC, (d) IPMN, (**e**) SCA, (**f**) SPT. From row1 to row4 are non-enhanced phase, arterial phase, portal phase, delayed phase

Our dataset are the real pancreas cancer CT images from the General Surgery Department of Peking Union Medical College Hospital. There are totally 59 patients, including 15 patients with non-pancreas diseases, and 44 with pancreas-related diseases, with a sum of 236 image slices. With the informed consent, patients’ information, including name, gender, age, are confidential. At the slice level, one patient has 4 abdomen CT images in different phases, such as non-enhanced phase, arterial phase, portal phase, delayed phase. Additionally, the five sorts of pancreas-related diseases included in the dataset are: PDAC (Pancreatic Ductal Adenocarcinoma), PNET (Pancreatic Neuroendocrine Tumors), IPMN (Intraductal Papillary Mucinous Neoplasia), SCA (Serous CystAdenoma of the pancreas), and SPT (Solid Pseudopapillary Tumour of the pancreas) (Fig. 1).

### Multi-layer up-sampling structure

#### Network architecture

Inspired by the previous work on deep convolutional neural network [17, 26], we design our network by modifying the RCF network [27]. Based on Holistically-nested Edge Detection (HED) network, it is an edge detection architecture aiming to extract visually salient edges and object boundaries from natural images [27].

**Fig. 2:**
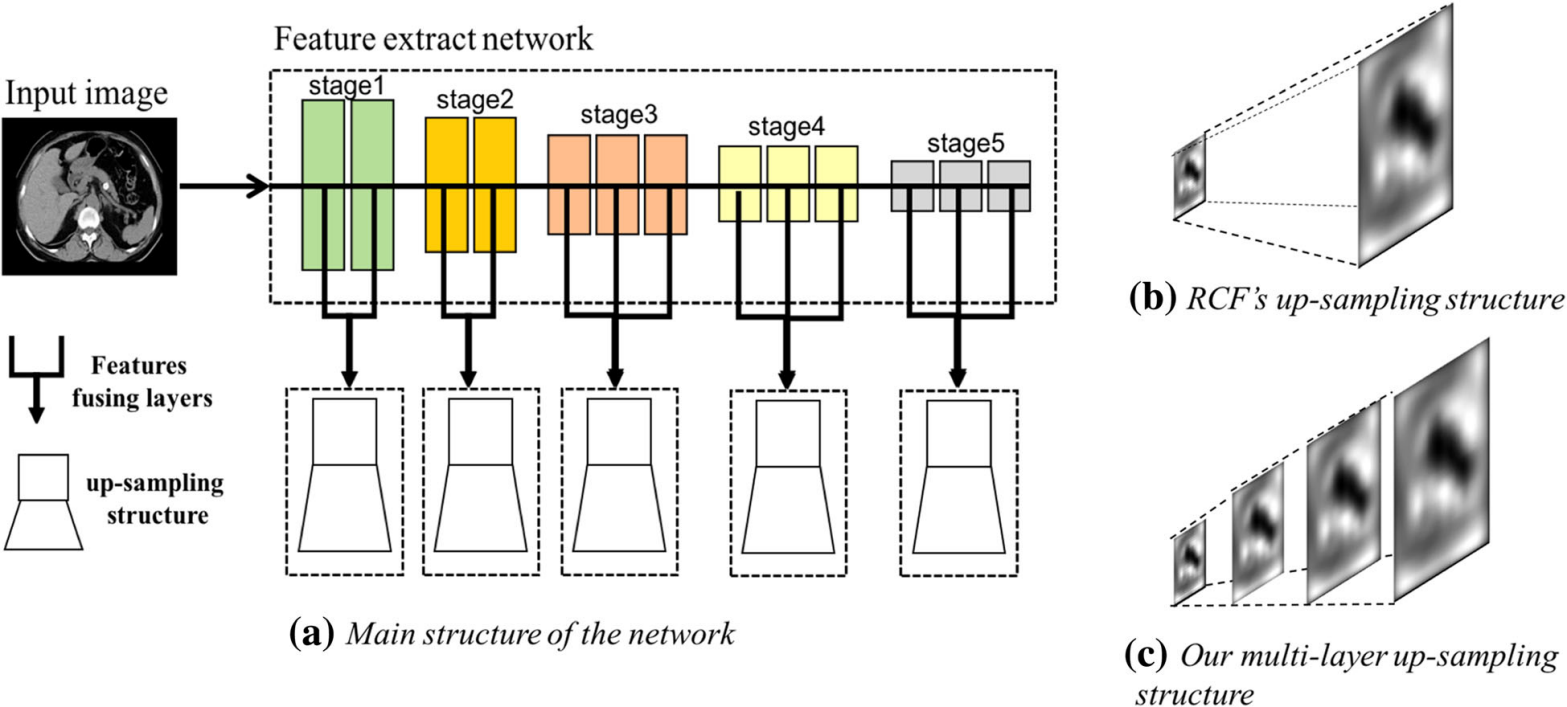
Architecture of our network. Part (**a**) shows the main structure of our network. In the feature extract network, each color box stands for a conv layer, and the conv layers are divided into 5 different stages in different colors. Furtherly, each stage is connected to a features fusing layer. After that, an up-sampling structure is used to de-convolute the extracted features to the initial size. Part (**b**) and (**c**) separately show the up-sampling structure of the RCF network and ours

The whole network contains a feature extraction network and 5 feature fusing layers with up-sampling layers. The feature extraction network contains 13 conv layers and 4 pooling layers [27], which are divided into 5 stages (shown in Fig. 2). Different from the traditional classification network, there is no fully connected layer in the network. Besides, to get richer interior information and improve the overall performance, the RCF network combines the hierarchical features extracted from the 5 stages of the convolutional layers.

Each stage combines a feature fusing layer, i.e., each convolutional layer in each stage is connected to a convolutional layer with kernel size 1*1 and channel depth 21 and then the resulting is accumulated using an element-wise layer to attain hybrid features [26], and a 1*1–1 convolutional layer follows them. After the feature fusing layer, an up-sampling structure (also called de-convolution) is used to up-sample the feature map to the input image size. Beneficial from the non-full-connection layers and up-sampling structures, the network can duel with input images in arbitrary size and output the response-size probability map.

In the up-sampling process, the images outputted by the last layer has to be resized as the input images, thus more detailed texture information is added into the images. The starting point of our network design lies in the construction of this detailed texture information.

The novel network proposed by us is shown in the part (a) of Fig. 2. Compared with RCF, our modifications can be described as following: We adopt the multi-layer up-sampling structures to replace the four de-convolutional layers. Then on the stage 2 to 5, the 1*1–1 conv layer is connected by the multi-layer up-sampling structure, and the output images of them are combined in the fusion stage.

Our novel structure consists of several up-sampling layers that include diverse convolutional kernels. We initialize them with bilinear interpolation. Then in the training process, the convolutional kernels in the layers continuously learn and adjust the parameters during iteration and repeated optimization.

Compared with the task of edge detection, single object segmentation requires the model containing far more accurate detailed texture information. In the previous RCF network, the de-convolutional layer could produce the loss pixels and resize the images, but resulting from the simple bilinear interpolation, the information added is too coarse to segment the object. As we all know, in an image, there are strong relationships between the neighbor pixels, and it is an ideal method to produce a missing pixel by using its nearest neighbors. However, adopting only one step of up-sampling may lead to produce a pixel by comparably far ones since too much pixels are missed in the images. In contrast, a multi-layer up-sampling structure ensures that a missing pixel is produced by its neighbors by multi-step up-sampling, and furtherly guarantees higher quality of output on each stage. Additionally, different from simple bilinear interpolation, the pattern, that the convolutional kernels adjust the parameters during the training process, assures the up-sampling operation and the whole model fit the local dataset better by producing a set of optimized parameters. The comparison of up-sampling structure in the RCF network and ours is shown in the part (b) and (c) in the Fig. 2.

**Fig. 3:**
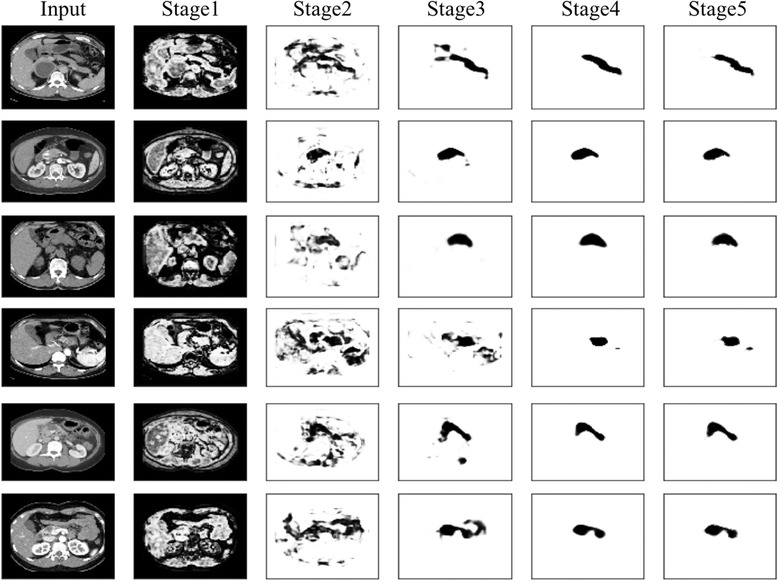
Example of multistage output. The first column is the original input from our datasets. And from row 1 to row 6 are the six classes of pancreas disease, namely healthy, PNET, PDAC, IPMN, SCA, SPT. From the column 2 to column 6 are the output of stage 1 to 5 from our model

Hence, we acquire multi-stage outputs with more accurate detailed texture information helpful to single object segmentation. We show the intermediate results from each stage in Fig. 3. Compared with the five outputs of RCF, they are obviously in higher quality. And the quantized advantages are shown in section 3.

#### Loss function

To train and optimize our segmentation model, we adopt per pixel loss function [26], and thus necessary to have the ground-truth maps. Each CT scan has been labeled by an annotator with medical knowledge. The ground-truth maps show the edge possibility of each pixel. 0 means that the annotator does not label at this pixel, and 1 means that the annotator labels at this pixel. Additionally, the negative sample consists of pixels with possibility value equal to 0, and the positive sample consists of other pixels.

1 $$ L(W)=\kern0.5em {\sum}_{i=1}^{\mid I\mid}\left({\sum}_{k=1}^Kl\left({X}_i^{(k)};W\right)+l\left({X}_i^{fuse};W\right)\right), $$

K means the number of stages making output. As shown in the Equation1, the loss value of each image is the addition of loss value of each pixel, which is made of loss value of each stage-out and fusion stage.$l\left({X}_i^{(k)};W\right)$ denotes the loss value of a pixel in the k-th stage. Similarly, $l\left({X}_i^{fuse};W\right)$ denotes the loss value of a pixel in the fusion stage. *X*_*i*_ is the activation value (feature vector) at pixel i. W is all the parameters in our network. |I| is the number of all pixel in an image.

2 $$ l\left({X}_i;\mathrm{W}\right)=\left\{\begin{array}{c}\alpha \ast \log \left(1-P\left({X}_i;W\right)\right)\  if\ {y}_i=0\\ {}\beta \ast \log P\left({X}_i;W\right)\kern2.5em otherwise\end{array}\right. $$

*P*(*X*_*i*_; *W*) is the edge possibility value at pixel i. P denotes the standard sigmoid function.

3 $$ \left\{\begin{array}{c}\upalpha =\uplambda \ast \frac{\left|{Y}^{+}\right|}{\left|{Y}^{+}\right|+\left|{Y}^{-}\right|}\\ {}\upbeta =\frac{\mid {Y}^{+}\mid }{\mid {Y}^{+}\mid +\mid {Y}^{-}\mid}\end{array}\right. $$

To balance the negative and positive sample, we adopt the hyper-parameter λ (λ is set as 1.1 when training). *Y*^+^ denotes the positive sample of an image, and *Y*^−^ denotes the negative sample of an image.

#### Workflow of our segmentation

**Fig. 4:**
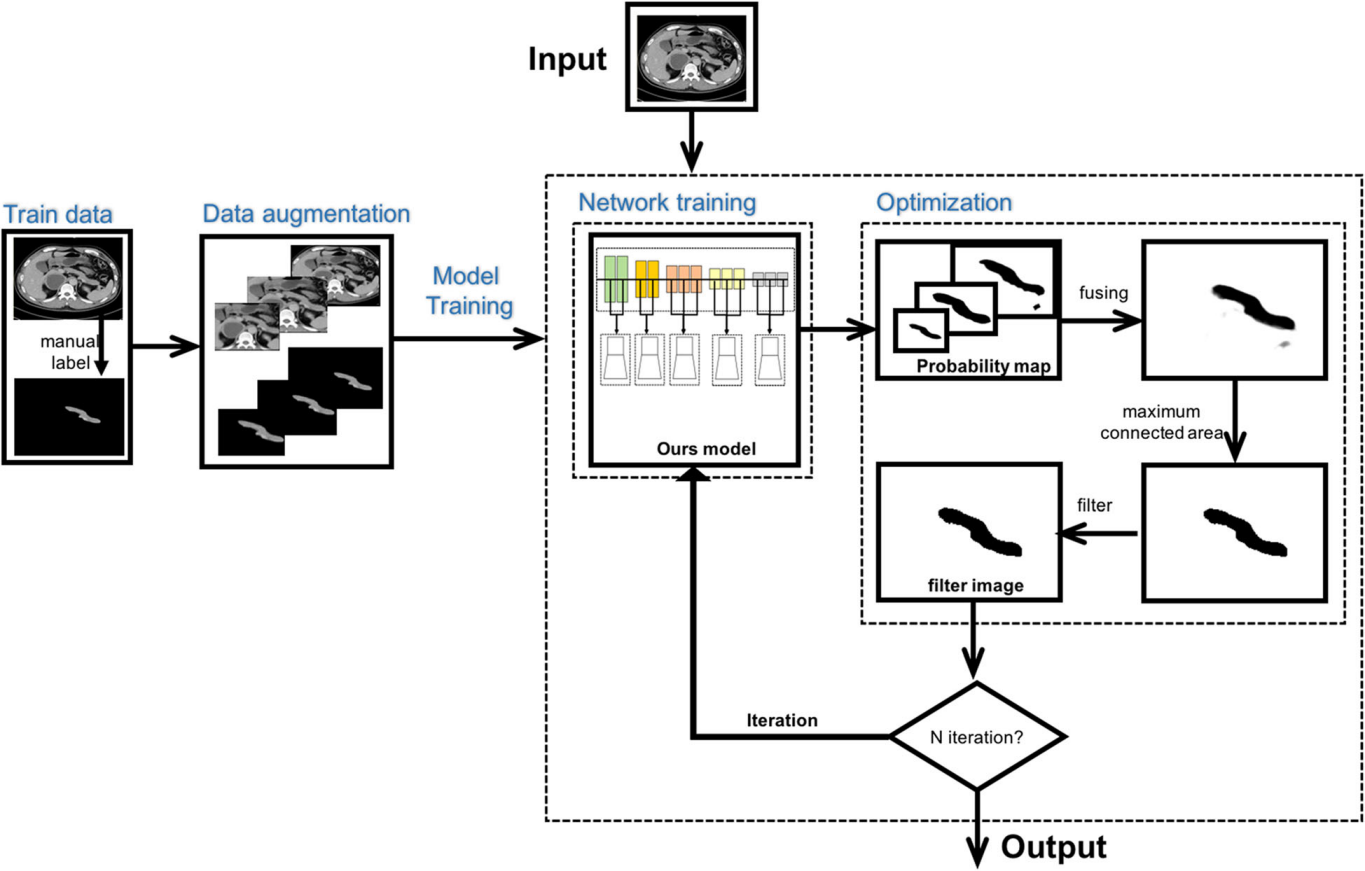
Workflow of the segmentation process. The data with manually label are used to training and optimization. When the whole architecture is trained, the architecture receives the input CT images and directly output the pancreas segmentation result

We implement a deep learning framework based on our new multi-layer up-sampling neural network for pancreas segmentation (Fig. 4). The segmentation pipeline consists of two modules, model training and optimization (Fig. 4).

In the model training module, firstly we preprocess both the original CT images and the ground truth images. The original images are in different size about 400 pixels*500 pixels. We resize the images’ height to 256 pixels and keep the ratio of each image’s height and width. Reducing the size of the images can not only speed the model training, but also retain more information of the original data.

After resizing the image size, to enlarge the training dataset and prevent the deep learning model over-fitting, we do the data augmentation basing on [28], such as translation transform and scale transform. After that, we trained our multi-layer up-sampling neural network based on Convolution Neural Network (CNN). Since the dataset is still small, we adopt transfer learning, i.e., fine-tuning our CNN models pre-trained from BSDS500 dataset [26] (a natural dataset for edge detection) to our medical CT image tasks, which [29] has examine why transfer learning from pre-trained natural dataset is useful in medical image tasks. After pre-training, the model gets an original set of parameters, and then was fine-tuned in our dataset, so that the network could easily converge in our dataset with a higher speed.

Our advanced model outputs a probability map of each training data. The probability map is in response-size with the input image, whose pixels are the probability of the corresponding pixel’s belonging to pancreas. Besides, to highlight the pancreas, we rescale the probability map from the grey [0, 1] to [0,255] and do the gray value inversion, so in the probability map, darker region has higher probability to be pancreas.

The optimization module is divided into 3 steps: fusing, maximum connected area and threshold filter. In the fusing step, a set of probability maps belonging to the same input image is fused into a new image. To predict a specific pixel, we simply count the probability maps with its probability larger than 0. Then the specific pixel of a fuse image is made up of the mean of true positive pixel. In the maximum connected area step, after transforming the fuse image to binary image, we search the fused image’s pixels to find the non-zeros neighbors of current pixel, and obtain one or several connected areas. Then we select the region with maximum area. In the filter step, we simply get a mask showing the maximum connected area, and use it to segment the pancreas from the original input image.

#### Evaluation criteria

Here, P is the prediction image, G is the ground-truth image, and S is the area of foreground in certain image. Then we have the following criteria:

**Precision** (also called positive predictive value), is the fraction of correctly predicted foreground area among that in prediction

4 $$ Precision=\frac{S\left(P\bigcap G\right)}{S(P)} $$

where S(P ⋂ G) is the interaction area in foreground of P and G.

**Recall** (also known as sensitivity), is the fraction of correctly predicted foreground area over that in ground-truth.

5 $$ Recall=\frac{S\left(P\bigcap G\right)}{S(G)} $$

**Dice Similarity Coefficient (DSC)**, measures the similarity of prediction image and ground-truth image. The definition of DSC is the same as *F1* score. Here we also give its relationship with precision and recall.

6 $$ {\displaystyle \begin{array}{l} DSC\left(P,G\right)=\frac{2^{\ast }S\left(P\cap G\right)}{S(P)+S(G)}=\kern0.5em \frac{2}{\frac{S(P)}{S\left(P\cap G\right)}+\frac{S(G)}{S\left(P\cap G\right)}}\\ {}\kern8.5em =\frac{2}{\frac{1}{precision}+\frac{1}{recall}}=\frac{2\ast precision\ast recall}{precision+ recall}\end{array}} $$

**Jaccard similarity coefficient,** also known as Intersection over Union (originally coined coefficient de communauté by Paul Jaccard), is a statistic used for comparing the similarity and diversity of prediction image and ground-truth image. It is defined as the size of the intersection area divided by the size of the union area:

7 $$ \mathrm{Jaccard}\left(\mathrm{P},\mathrm{G}\right)=\frac{S\left(P\bigcap G\right)}{S\left(P\bigcup G\right)}=\frac{S\left(P\bigcap G\right)}{S(P)+S(G)-S\left(P\bigcap G\right)} $$

All of the criterias ranges from 0 to 1, with best value at 1 and worst at 0.

## Results

In our experiment, we randomly split the dataset of 59 patients into 5-folds, training and testing folds, with 10, 10, 10, 10 and 9 for each one. Then we do data augmentation, such as zooming in, flipping, rotating for each training data and enlarge the data into 128 times, and the whole dataset up to 30,208 images.

Besides, our CNN model is pre-trained in BSDS500 dataset and fine-tuned in our dataset with stochastic gradient descent (SGD) algorithm and step-wise learning schedule to optimize. The model is implemented by a deep learning framework CAFFE [30] and run over one NVIDIA QUADRO M4000 GPU.

Using 5-fold cross-validation, we could achieve a mean of precision of 76.83%, a mean of recall of 78.74%, a mean of DSC of 75.92%, and the mean of JACCARD of 63.29%. Apart from the recall one, all of them are higher than the RCF network. At the same time, our method with multi-scale input (OURS-MS) reaches 77.36%, 79.12%, 76.36%, 63.72% in mean of precision, recall, DSC and Jaccard. Table 1 show the detailed performance of three models.

**Table 1:** Compare the three segmentation models’ performance in four measurements: precision, recall, DSC and Jaccard index

	PRECISION		RECALL		DSC		JACCARD	
	mean	Std	mean	Std	mean	Std	mean	Std
rcf	74.35	18.97	79.83	16.70	74.91	15.25	61.98	17.44
ours	76.83	18.53	78.74	17.19	75.92	15.17	63.29	17.45
ours-ms	77.36	17.96	79.12	16.27	76.36	14.34	63.72	17.05

**Fig. 5:**
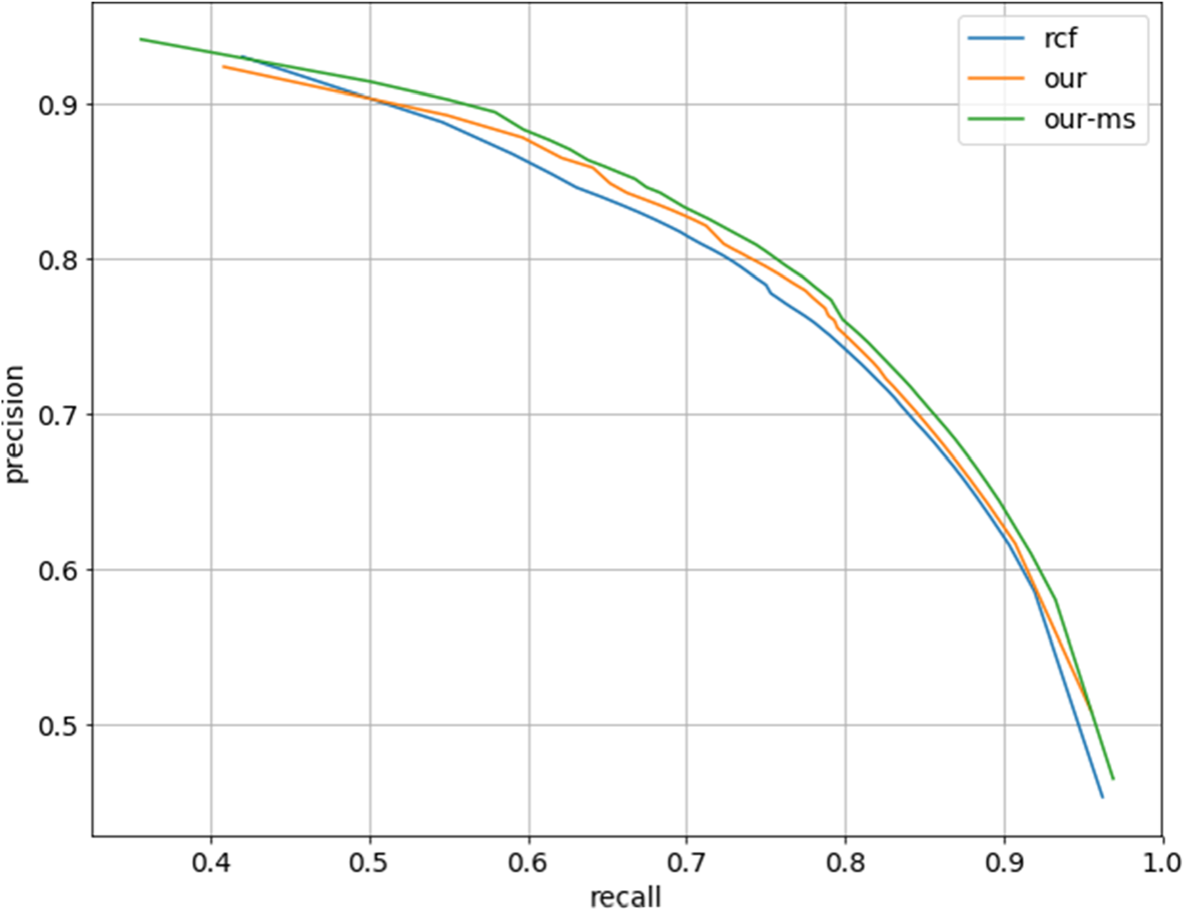
The Precision-Recall curve. The blue, orange and green curves stand for the performance of RCF, our model and OURS-MS.

In the pancreas segmentation task, the number of positive samples is much less than that of negative samples, which means that the Precision-Recall (PR) curve can better reflect the performance of the prediction [31]. Figure 5 shows the Recall value can reach more than 90% while the Precision value is still more than 60%, which means that we could attain excellent reservation of the pancreas organ area in a decent precision.

Our model’s performances in different types of pancreas cancer are shown in Table 2. We can see that the values of four measurements are comparably high, and the standard deviations are not too big, which means that our model is robust in different types of pancreas cancer.

**Table 2:** Model’s performance in different types of pancreas cancer (with healthy type)

	PRECISION		RECALL		DSC		JACCARD	
	mean	Std	mean	Std	mean	Std	mean	Std
Healthy	80.95	14.94	86.53	9.03	82.41	9.45	71.10	12.73
PNET	75.39	21.06	67.37	21.08	68.95	18.45	55.26	19.26
PDAC	76.22	18.83	82.50	12.52	77.08	12.61	64.29	15.63
IPMN	70.44	22.42	74.86	22.42	71.90	21.71	60.04	24.04
SCA	80.51	9.76	83.62	8.90	81.51	7.03	69.38	10.28
SPT	75.03	21.47	74.72	14.28	71.45	11.49	56.81	14.43

Our model’s performances in different phases are shown in Table 3. We can see that the values of four measurements are comparably high, and the standard deviations are not too big, which means that our model is robust in different phases.

**Table 3:** Model’s performance in different phases. The Phase1 to Phase4 are non-enhanced phase, arterial phase, portal phase, delayed phase

	PRECISION		RECALL		DSC		JACCARD	
	Mean	std	mean	std	mean	std	mean	std
Phase1	80.22	18.26	79.78	16.57	78.04	15.40	66.29	18.59
Phase2	76.68	20.45	77.86	20.33	75.43	18.03	63.19	18.76
Phase3	74.64	18.52	78.61	15.04	74.71	13.56	61.36	16.18
Phase4	76.46	16.79	78.82	15.84	75.91	13.00	62.84	16.16

**Fig. 6:**
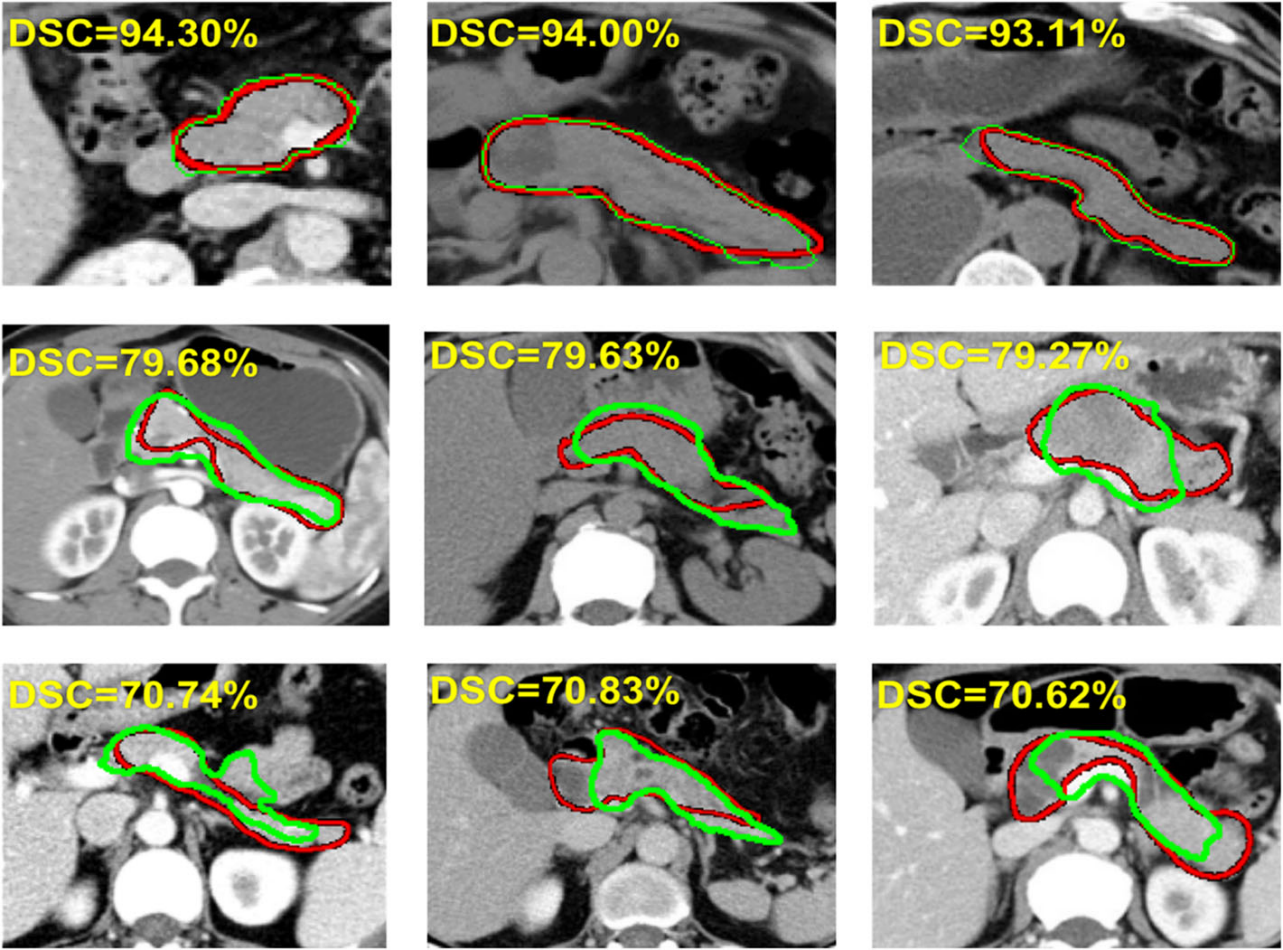
Some examples of pancreas segmentation result. Red curve shows the ground truth while green for the predicted. Row1 are in the best performance, row2 are on the quartile2 and row3 on the quartile1

Figure 6 shows some examples of the pancreas segmentation result, a comparison of ground-truth and output of our model. The red curve is ground-truth annotation, and the green curve highlights the output. We can easily find that the two curves of four images share high similarity, and high accuracy has been gained in our model. Images in row1 get the best performance, where the DSC values are around 94%, images in row2 get the DSC value on quartile2, around 79%, and those in row3 reach the DSC values around 70%, which is on the quartile1.

## Conclusions

We summarize our contributions as follow. In this paper, we design an automatically pancreas segmentation architecture based on deep learning model, and get a 76.36% DSC value.

We extend the Richer Convolutional Feature network to pancreas segmentation and improve the RCF network with multi-layer up-sampling structure and get over 1% better performance in pancreas segmentation. Besides, we find that, in experiment, testing with multi-scale input and training with data augmentation, especially rotation, can improve the performance of the network.

Significantly, our model is robust in different types of pancreas cancer and different phases of CT images.

## Abbreviations

CAD: Computer-Aided Diagnosis
CNN: Convolutional Neural Networks
CT: Computed Tomography
DSC: Dice Similarity Coefficient
FCN: Fully Convolution Network
HED: Holistically-nested edge detection
IPMN: Intraductal Papillary Mucinous Neoplasia
MALF: Multi-Atlas Registration & Label Fusion
PDAC: Pancreatic Ductal Adenocarcinoma
PNET: Pancreatic Neuroendocrine Tumors
PR: Precision-Recall
RCF: Richer Convolutional Feature network
SCA: Serous CystAdenoma of the pancreas
SGD: stochastic gradient descent
SPT: Solid Pseudopapillary Tumour of the pancreas
Std: Standard deviation
